# Surgery of the Spine

**Published:** 1918-08

**Authors:** 


***Military Aspects of the Surgery of the Spine and Spinal Cord.*** By CHARLES H. FRAZIER, *Surgery, Gynecology and Obstetrics,* June 1918.

This paper was read before the Clinical Congress of Surgeons of North America on October 24, 1917 by Dr. Frazier who is conducting with others a course in neurological surgery for members of the Medical Reserve Corps of the U. S. Army. The technic of the usual laminectomy is well illustrated and its general features are unchanged. A few new details in refinement of technic have been introduced. The clinical part of the paper is based on the excellent observations of Gordon Holmes and this is concerned with localization of the injury in the cord. There appear to be four groups of cases which are determined by the nature, the seat, and the extension of the lesion.

Group 1. Comprises complete transverse lesions with absolute flaccid paralysis below level of injury, abolition of all reflexes and all sensations.

Group 2. Comprises partial lesions; spinal hemiplegias, more or less typical Brown Sequard syndromes.

Group 3. Comprises lesions of compression producing spastic paraplegia, exaggerated reflexes, and positive Babinski.

Group 4. Comprises lesions of the cauda equina.

In localizing the lesion, motor palsies of isolated muscles or muscle groups, including those of the trunk, are even more valuable than the sensory disturbances. Lesions between the second cervical and second thoracic segments are liable to have sympathetic symptoms, i. e., myosis or inequality of the pupils, narrowing of the palpebral fissure; enopthalmos, diminished tear secretion; flushing of the face, diminished sweating and dryness of the affected side.

Lesion of the lower part of the cervical enlargement may show slow pulse, low blood pressure, and scanty urine. In involvement of dorsal segments the corresponding intercostal muscle or muscles may be paralyzed as determined by palpation of the intercostal space during the usual inspiratory contraction. In involvement of the ninth dorsal, the lower half of the rectus abdominis is paralyzed. In involvement of the 11th dorsal segment there is bulging of the external and internal obliquus over the iliac fossae, but the rectus is not paralyzed.

As to indications for operation, Frazier is very conservative. In direct injuries to the cord he says the propriety of laminectomy cannot be discussed; this, whether the symptoms indicate a complete or incomplete lesion. Oppenheim also believes operation is indicated even in cases of total transverse lesion, for there is nothing to lose and perhaps something to be gained. In indirect injuries, those due to explosion in the vicinity, or to impact of a bullet against the vertebral column without direct impact on the cord, early operation is contra-indicated according to Frazier.

